# Plasma LTA4H as an Inflammatory Target for Monitoring +Gz-Induced Injury in Rabbits

**DOI:** 10.3390/cimb48100982

**Published:** 2026-09-24

**Authors:** Yunchong Wu, Tailai Luo, Jia Zhai, Yihe Wang, Lichao Wang, Yutao He, Jun Shen, Yongjie Yao, Wei Zhu

**Affiliations:** Naval Medical Center, Naval Medical University, Shanghai 200433, China; yunchong_wu@163.com (Y.W.); lambltl@smmu.edu.cn (T.L.);

**Keywords:** hypergravity, LTA4H, LTB4, IL-6, inflammation, biomarker

## Abstract

Repeated +Gz exposure during high-performance flight can cause inflammatory injury, but whether LTA4H and its related molecules are altered after such exposure and whether LTA4H could serve as a plasma target for monitoring +Gz-induced inflammatory injury remain unclear. Eighteen male New Zealand white rabbits were assigned to control, 2-week, and 4-week groups (*n* = 6 per group). Plasma proteomics was performed using data-independent acquisition mass spectrometry. LTA4H, LTB4, and IL-6 were quantified by ELISA. Correlations among the three molecules were analyzed by Pearson correlation. Diagnostic performance was assessed by receiver operating characteristic curve analysis. Proteomics identified LTA4H as nominally upregulated in both the 2-week and 4-week groups, but it did not remain significant after false discovery rate correction. It was therefore selected as an exploratory candidate for orthogonal confirmation by ELISA. ELISA confirmed that plasma LTA4H, LTB4, and IL-6 were all significantly elevated after +Gz exposure. Positive correlations were observed in the pooled analysis, but these were attenuated after adjusting for group. LTA4H showed exploratory discrimination of exposure status, with an AUC exceeding 0.9. Repeated +Gz exposure was associated with concurrent elevation of LTA4H, LTB4, and IL-6. LTA4H may serve as a potential plasma target for monitoring +Gz-induced inflammatory injury.

## 1. Introduction

High-performance fighter aircraft during aerial combat maneuvers generate +Gz characterized by high G magnitude, rapid onset rate, prolonged duration, and repeated exposure. The resultant acceleration overload can substantially exceed physiological tolerance limits, inflicting mechanical stress and hemodynamic perturbations on multiple organ systems [1,2,3]. Blood volume redistribution toward the lower body and splanchnic vascular beds, combined with reduced effective circulating volume, leads to a combined congestive and hypoxic insult with documented ischemia–reperfusion injury in the liver, heart, and other organs [4,5]. This process is accompanied by inflammatory responses, yet circulating biomarkers that can monitor such +Gz-induced inflammatory injury remain lacking.

Ischemia–reperfusion injury activates the arachidonic acid cascade and its downstream 5-lipoxygenase pathway, which generates pro-inflammatory lipid mediators [6]. Leukotriene A4 hydrolase (LTA4H) is a critical enzyme in this pathway. It catalyzes the conversion of leukotriene A4 (LTA4) into leukotriene B4 (LTB4), a potent chemoattractant that drives neutrophil recruitment and amplifies inflammation [7,8]. LTB4 can stimulate the production of inflammatory cytokines such as interleukin-6 (IL-6) [9]. IL-6 has also been associated with enhanced LTA4H synthesis and release [10]. These observations suggest a potential interaction among LTA4H, LTB4, and IL-6 in inflammatory conditions, although direct causal evidence was not obtained in this study. LTA4H has been investigated as a biomarker in chronic inflammatory conditions [11], but its expression and diagnostic value in the context of +Gz-induced injury have not been explored.

In this study, we used an untargeted DIA proteomic approach to screen for differentially abundant plasma proteins in rabbits subjected to repeated +Gz exposure. We focused on LTA4H and measured its downstream product LTB4 and upstream cytokine IL-6 by ELISA. We then examined correlations among the three molecules and assessed the exploratory discriminative performance of LTA4H by ROC curve analysis. Our goal was to explore whether LTA4H could serve as a potential plasma target for monitoring +Gz-induced inflammatory injury.

## 2. Materials and Methods

### 2.1. Reagents and Instruments

ELISA kits for rabbit LTA4H (catalog no. YC-1107T2) and IL-6 (catalog no. YC-0302O2) were purchased from Shanghai Yuanchuang Biotechnology Co., Ltd. (Shanghai, China). The ELISA kit for rabbit LTB4 (catalog no. JM-00720R2) was obtained from Jingmei Biotechnology Co., Ltd. (Yancheng, China). The animal centrifuge (model GSF-2) was a custom-built device provided by the Naval Medical Center, Naval Medical University (Shanghai, China).

### 2.2. Animals and Experimental Groups

Eighteen healthy male New Zealand white rabbits (3–6 months old, body weight 2.5–3.5 kg) were obtained from the Laboratory Animal Center of the Naval Medical Center [license no. SCXK (Shanghai) 2023-0009] (Shanghai, China). The rabbits were randomly assigned to three groups (*n* = 6 per group): a control group, a 2-week group, and a 4-week group. All animals were housed individually in stainless steel cages under controlled environmental conditions with a temperature of 25 °C, humidity of 50–60%, and a 12-h light/dark cycle. They had free access to standard rabbit chow and water. No environmental enrichment was provided. Animals were acclimatized for 1 week before the experiment began. Rabbits in the control group were restrained in the same 3D-printed holders as the exposure groups and placed beside the centrifuge for the same duration, experiencing comparable restraint and noise stress but without centrifugation. Only male rabbits were used, consistent with the predominantly male fighter pilot population and the conventions of aeromedical research. The study protocol was approved by the Medical Ethics Committee of the Naval Medical Center (approval no. 2026H002; date of approval: 11 March 2026), and all animal procedures were performed in accordance with the institutional guidelines for animal experimentation. The control group underwent the same restraint and noise stress for 28 consecutive days and was euthanized at the same endpoint as the 4-week group.

### 2.3. +Gz Exposure Protocol

The animal centrifuge had an arm length of 2.0 m and generated a +Gz acceleration environment via centrifugation. Each rabbit was restrained in a custom-made 3D-printed holder and fixed on the rotating platform with the head oriented toward the center and the tail toward the periphery. The +Gz exposure profile consisted of a target +6 Gz, a G-onset rate of 1 G/s, and a plateau duration of 60 s per exposure. Exposures were repeated six times daily with a 10-min interval between successive runs. The 2-week group underwent 14 consecutive days of this protocol, and the 4-week group underwent 28 consecutive days [12]. Humane endpoints were predefined in the ethics protocol. Any animal showing paralysis or other severe distress would be euthanized immediately to prevent unnecessary suffering.

### 2.4. Sample Collection and Processing

On the day after the final exposure session, approximately 24 h after the last exposure, rabbits were deeply anesthetized with 5% isoflurane (RWD Life Science, Shenzhen, China). Blood samples were collected from the marginal ear vein into EDTA-anticoagulant tubes (BD Vacutainer, Becton Dickinson, Franklin Lakes, NJ, USA). Under deep anesthesia, animals were euthanized, and death was confirmed by the absence of spontaneous respiration and heartbeat. Blood samples were centrifuged at 1300× *g* for 10 min at 4 °C, and the supernatant plasma was aliquoted into cryovials, snap-frozen in liquid nitrogen, and stored until analysis. 

### 2.5. Plasma Proteomic Profiling

Plasma samples from the three groups were subjected to high-throughput data-independent acquisition (DIA) proteomic analysis using an Astral mass spectrometer (Thermo Orbitrap Astral; Thermo Fisher Scientific, Waltham, MA, USA) at Shanghai Majorbio Bio-Pharm Technology Co., Ltd. (Shanghai, China). High-abundance proteins were depleted using the EasyPept-DeeP Low-Abundance Protein Enrichment Kit (Shanghai Omicsolution Co., Ltd., Shanghai, China). Protein identification and quantification were performed using Spectronaut software version 20 (Biognosys AG, Schlieren, Switzerland) against the UniProt rabbit database (uniprot-taxonomy_9986_unique.fasta). The false discovery rate was set to 1% at both the peptide and protein levels. Proteins with missing values in more than 70% of all samples were removed. Missing values were then imputed using the K-nearest neighbors method within each group, provided that the protein was detected in at least 30% of samples in any group. Data normalization was performed automatically by Spectronaut, which selects between global and local normalization based on the number of runs. Protein-level *p* values were obtained using Spectronaut’s default statistical workflow.

### 2.6. Selection of Differentially Abundant Proteins

Differentially abundant proteins were filtered using the criteria |log_2_ fold change| ≥ 1 and nominal *p* < 0.05. Multiple testing correction was performed using the Benjamini–Hochberg method, and adjusted *p* values were calculated. The complete list of differentially abundant proteins is provided in Supplementary Table S1.

### 2.7. ELISA Assays

Plasma levels of LTA4H, IL-6, and LTB4 were measured using commercial rabbit ELISA kits according to the manufacturers’ instructions Supplementary Table S2. The LTA4H and IL-6 kits used a double-antibody two-step sandwich method, with sensitivities of 0.625 ng/mL and 5 pg/mL, and detection ranges of 0.625–10 ng/mL and 5–80 pg/mL, respectively. The LTB4 kit used a double-antibody one-step sandwich method, with a sensitivity of 1 pg/mL and a detection range of 1–200 pg/mL. All plasma samples were diluted fivefold before measurement. The optical density was read at 450 nm, and concentrations were calculated from a standard curve using linear regression. The intra-assay and inter-assay coefficients of variation were below 15% for all three kits. All measurements were performed in duplicate, and the mean value of the duplicates was used for statistical analysis.

### 2.8. Statistical Analysis

All statistical analyses were performed using GraphPad Prism version 10.1.2 (GraphPad Software, San Diego, CA, USA). Prior to parametric testing, the normality of data distribution was assessed using the Shapiro–Wilk test. Homogeneity of variance was assessed using Levene’s test. Differences in biomarker concentrations among the three groups were assessed by one-way analysis of variance followed by Tukey’s multiple comparisons test. To account for group differences, multivariable linear regression was performed with each biomarker as the dependent variable and group and the other two biomarkers as independent variables. Variance inflation factors were calculated to assess collinearity. The discriminative performance of LTA4H in distinguishing exposed animals from controls was evaluated using receiver operating characteristic curve analysis. The optimal cut-off value was determined by maximizing the Youden index. Pearson correlation analysis was used to examine the associations among plasma LTA4H, LTB4, and IL-6 levels. All tests were two-sided, and *p* < 0.05 was considered statistically significant. The sample size was based on previous studies using the same +Gz exposure model [12]. No formal a priori sample-size calculation was performed. The investigators were not blinded to group allocation during the experiment or data analysis.

## 3. Results

### 3.1. Identification and Orthogonal Confirmation of Plasma LTA4H After Repeated +Gz Exposure

Plasma proteomic profiling was performed using DIA mass spectrometry. Using the screening criteria of |log_2_ fold change| ≥ 1 and *p* < 0.05, a total of 304 differentially abundant proteins were identified in the 2-week group compared with controls, of which 196 were upregulated and 108 downregulated. In the 4-week group, 229 differentially abundant proteins were identified, with 57 upregulated and 172 downregulated. LTA4H showed nominal upregulation in both the 2-week and 4-week groups (2 weeks: nominal *p* = 0.001, adjusted *p* = 0.077; 4 weeks: nominal *p* = 0.015, adjusted *p* = 0.285). After Benjamini–Hochberg correction, LTA4H did not reach statistical significance. However, it showed a consistently large fold change in both comparisons (|log_2_ fold change| = 3.22 at 2 weeks and 2.32 at 4 weeks) and is a key enzyme in the leukotriene inflammatory pathway. It was therefore selected as an exploratory candidate for orthogonal confirmation by ELISA. (Figure 1A,B).

ELISA was used to orthogonally confirm the proteomic results. Plasma LTA4H levels were significantly elevated in the 2-week group (*p* = 0.0002) and the 4-week group (*p* = 0.010) relative to the control group. The two exposure groups did not differ significantly from each other (Figure 1C).

### 3.2. LTB4 and IL-6 Are Elevated in Plasma After +Gz Exposure

LTA4H converts LTA4 to the potent chemoattractant LTB4, and it has been associated with inflammatory cytokines such as IL-6. We therefore measured plasma LTB4 and IL-6 by ELISA. Both were significantly elevated after +Gz exposure. LTB4 increased in the 2-week group (*p* = 0.0009) and rose further in the 4-week group (*p* < 0.0001), with a significant difference between the two exposure groups (*p* = 0.016) (Figure 2A). IL-6 also increased significantly, at both 2 weeks (*p* < 0.0001) and 4 weeks (*p* = 0.0017), again with a significant difference between the two exposure groups (*p* = 0.038) (Figure 2B).

### 3.3. LTA4H, LTB4, and IL-6 Are Concurrently Elevated After +Gz Exposure

To examine whether the elevations in LTA4H, LTB4, and IL-6 were interrelated, Pearson correlation analysis was performed. All three pairwise comparisons showed significant positive correlations. The strongest correlation was between LTA4H and IL-6 (*r* = 0.76, *p* = 0.0003), followed by LTB4 and IL-6 (*r* = 0.49, *p* = 0.038), and LTA4H and LTB4 (*r* = 0.47, *p* = 0.047) (Figure 3).

To account for group differences, multivariable linear regression was performed with each biomarker as the dependent variable and group and the other two biomarkers as independent variables. The models were significant overall (IL-6: *F* = 11.94, *p* = 0.0003, *R*^2^ = 0.786; LTB4: *F* = 14.55, *p* < 0.0001, *R*^2^ = 0.817; LTA4H: *F* = 6.906, *p* = 0.0033, *R*^2^ = 0.680). Group effects were significant for IL-6 and LTB4, but not for LTA4H. After adjusting for group, none of the pairwise associations among LTA4H, LTB4, and IL-6 remained statistically significant (all *p* > 0.05).

Taken together, these results indicate that the three molecules increased concurrently after +Gz exposure, but the present study does not provide evidence for a direct causal cascade among them. The full multivariable regression results are provided in Supplementary Table S3.

### 3.4. LTA4H Shows Strong Discriminative Performance

ROC curve analysis was performed to evaluate the ability of LTA4H to distinguish +Gz-exposed animals from controls. Because LTA4H levels did not differ between the 2-week and 4-week groups, the two groups were also combined for analysis. The AUC was 1.000 (95% CI: 1.000–1.000) for the 2-week group, 0.917 (95% CI: 0.757–1.000) for the 4-week group, and 0.958 (95% CI: 0.874–1.000) for the combined exposure group. Sensitivity and specificity were 1.000 and 1.000 at 2 weeks, 0.667 and 1.000 at 4 weeks, and 0.833 and 1.000 for the combined group (Figure 4). These results should be interpreted as exploratory discrimination of exposure status rather than diagnostic confirmation of injury. The optimal cut-off value was 8.06 ng/mL at 2 weeks and 7.70 ng/mL for both the 4-week and combined groups. Detailed parameters are provided in Supplementary Table S4.

## 4. Discussion

In this study, we identified plasma LTA4H as a consistently elevated protein after repeated +Gz exposure using DIA proteomics and orthogonal analytical confirmation by ELISA. Its downstream product LTB4 and upstream cytokine IL-6 were also significantly elevated. The three molecules showed positive correlations in the pooled analysis, but these associations were attenuated after adjusting for group. The strongest correlation was between LTA4H and IL-6. These findings suggest that repeated +Gz exposure is associated with concurrent elevation of LTA4H, LTB4, and IL-6, which may reflect a coordinated inflammatory response.

Repeated +Gz exposure is known to cause vascular and tissue stress, blood flow redistribution, and ischemia–reperfusion injury in multiple organs. Under +Gz acceleration, blood volume shifts toward the lower body and splanchnic vascular beds, reducing effective circulating volume and creating a combined congestive and hypoxic insult [13,14]. Previous studies have shown that repeated +Gz exposure triggers myocardial inflammatory responses in rats [15], and that mechanically induced tissue ischemia–reperfusion can lead to substantial inflammatory injury [6]. Ischemia–reperfusion can activate the arachidonic acid cascade and its downstream 5-lipoxygenase pathway, leading to the production of pro-inflammatory lipid mediators. LTA4H is a critical enzyme in this pathway, converting LTA4 into LTB4 [7,8]. LTB4 is a potent chemoattractant that drives neutrophil recruitment and amplifies inflammatory responses [16]. Previous studies have shown that LTB4 promotes inflammation in multiple cell types, including macrophages and hepatocytes [17], and that deficiency of 5-lipoxygenase, the upstream enzyme responsible for LTB4 synthesis, reduces hepatic inflammation and hepatocyte damage [18]. LTB4 can stimulate the production of inflammatory cytokines such as IL-6 [10]. IL-6 has also been associated with enhanced LTA4H synthesis and release [9]. These observations suggest a potential interaction among LTA4H, LTB4, and IL-6 in inflammatory conditions. Our data are consistent with this model, but they do not establish causality.

To account for group differences, multivariable linear regression was performed. The models were significant overall, and group effects were significant for IL-6 and LTB4. However, after adjusting for group, none of the pairwise associations among LTA4H, LTB4, and IL-6 remained statistically significant. This indicates that the concurrent elevation of these molecules is mainly driven by exposure time rather than by direct interactions among them. The pooled correlations should therefore be interpreted with caution. The full regression results are provided in Supplementary Table S3.

LTA4H is a bifunctional enzyme with both hydrolase and aminopeptidase activities and is expressed in multiple cell types [19,20]. In the context of +Gz-induced stress, circulating LTA4H may be released from multiple cellular sources. The exact origin could not be determined in this study, but this does not diminish the value of LTA4H as a potential monitoring target. Future studies using cell-specific approaches will be needed to clarify the sources of circulating LTA4H under +Gz stress.

ROC analysis showed that plasma LTA4H had an AUC of 1.000 at 2 weeks, 0.917 at 4 weeks, and 0.958 for the combined exposure group. These results should be interpreted as exploratory discrimination of exposure status, not as diagnostic confirmation of injury. As a plasma-based marker, LTA4H offers practical advantages for minimally invasive monitoring. Several limitations should be noted. The sample size was small and no independent validation cohort was included. Only male rabbits were used, consistent with the predominantly male pilot population, but generalizability requires further study. The cellular sources of circulating LTA4H were not identified. Other inflammatory markers were not measured for comparison. Humane endpoints were predefined in the ethics protocol, and no animals met the criteria for euthanasia during the study. The single control group was maintained for 28 days and therefore provides a time-matched control for the 4-week group but not a perfectly time-matched control for the 2-week group. In addition, only a single terminal measurement was obtained, and longitudinal sampling was not performed. Future studies should include larger independent cohorts, both sexes, and longitudinal sampling to further validate LTA4H as a potential monitoring target for +Gz-related injury.

## 5. Conclusions

In summary, this study provides the first evidence that repeated +Gz exposure is associated with concurrent elevation of LTA4H, LTB4, and IL-6 in rabbits. Plasma LTA4H was consistently elevated after +Gz exposure, and its upstream cytokine IL-6 and downstream product LTB4 were also significantly increased. The three molecules showed positive correlations in the pooled analysis, but these associations were attenuated after adjusting for group. LTA4H showed exploratory discrimination of exposure status. These findings suggest that LTA4H may serve as a potential plasma target for monitoring +Gz-induced inflammatory injury. Further validation in independent cohorts and elucidation of the cellular sources of LTA4H are warranted.

## Figures and Tables

**Figure 1:**
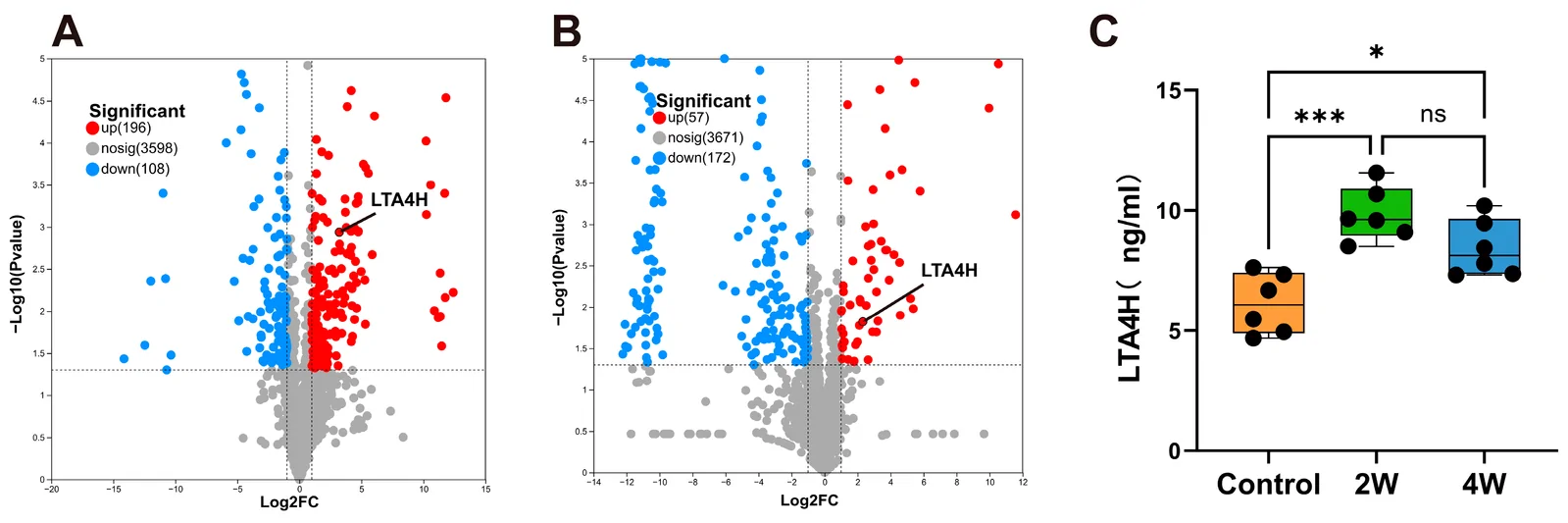
Identification and orthogonal confirmation of plasma LTA4H after repeated +Gz exposure. (**A**,**B**) Volcano plots showing differentially abundant proteins in the 2-week group versus control (**A**) and the 4-week group versus control (**B**). Proteins meeting the nominal screening criteria (|log2 fold change| ≥ 1 and nominal *p* < 0.05) are highlighted in red (upregulated) and blue (downregulated). LTA4H is indicated by arrows. After Benjamini–Hochberg correction, LTA4H did not reach statistical significance (adjusted *p* = 0.077 at 2 weeks and 0.285 at 4 weeks). (**C**) Plasma LTA4H levels by ELISA. Data are shown as box plots with all individual data points (*n* = 6 per group). The box represents the interquartile range, the horizontal line within the box indicates the median, and the whiskers extend to the minimum and maximum values. Statistical comparisons were performed by one-way ANOVA followed by Tukey’s multiple comparisons test. * *p* < 0.05, *** *p* < 0.001; ns, not significant.

**Figure 2:**
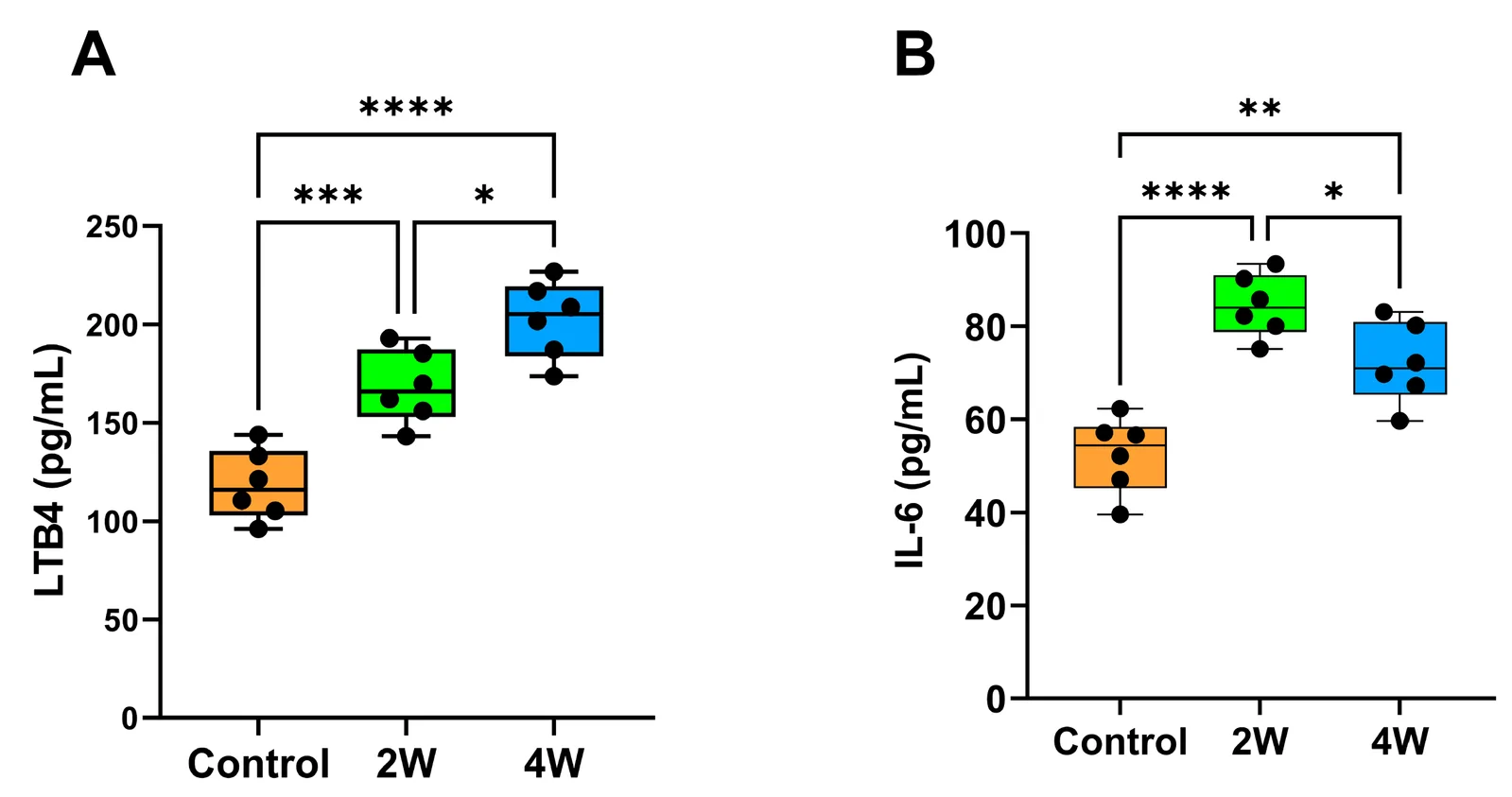
Elevation of plasma LTB4 and IL-6 after repeated +Gz exposure. (**A**) Plasma LTB4 levels by ELISA. (**B**) Plasma IL-6 levels by ELISA. Data are shown as box plots with all individual data points (*n* = 6 per group). The box represents the interquartile range, the horizontal line within the box indicates the median, and the whiskers extend to the minimum and maximum values. Statistical comparisons were performed by one-way ANOVA followed by Tukey’s multiple comparisons test. * *p* < 0.05, ** *p* < 0.01, *** *p* < 0.001, **** *p* < 0.0001.

**Figure 3:**
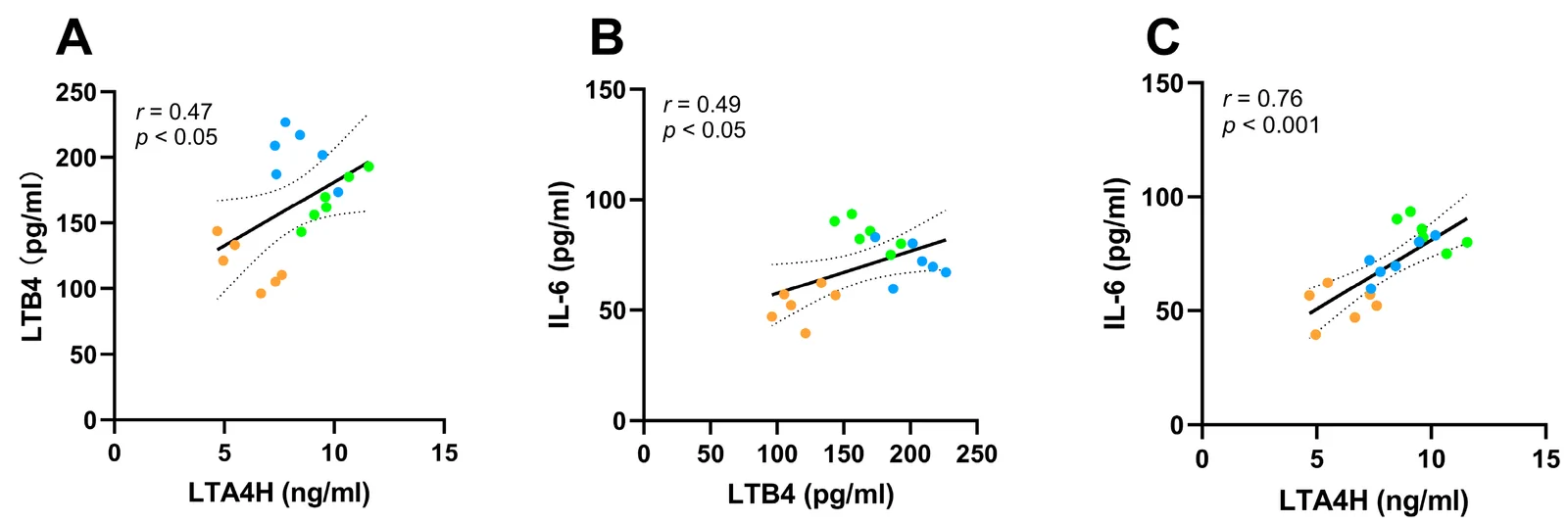
Correlation analysis among plasma LTA4H, LTB4, and IL-6. Pearson correlation scatter plots. Data points are color-coded by group: yellow for the control group, green for the 2-week group, and blue for the 4-week group. The solid line in each panel represents the linear regression fit. (**A**) LTA4H vs. LTB4: *r* = 0.47, *p* = 0.047. (**B**) LTB4 vs. IL-6: *r* = 0.49, *p* = 0.038. (**C**) LTA4H vs. IL-6: *r* = 0.76, *p* = 0.003. These are pooled correlations across all groups. Group-adjusted associations are reported in the text.

**Figure 4:**
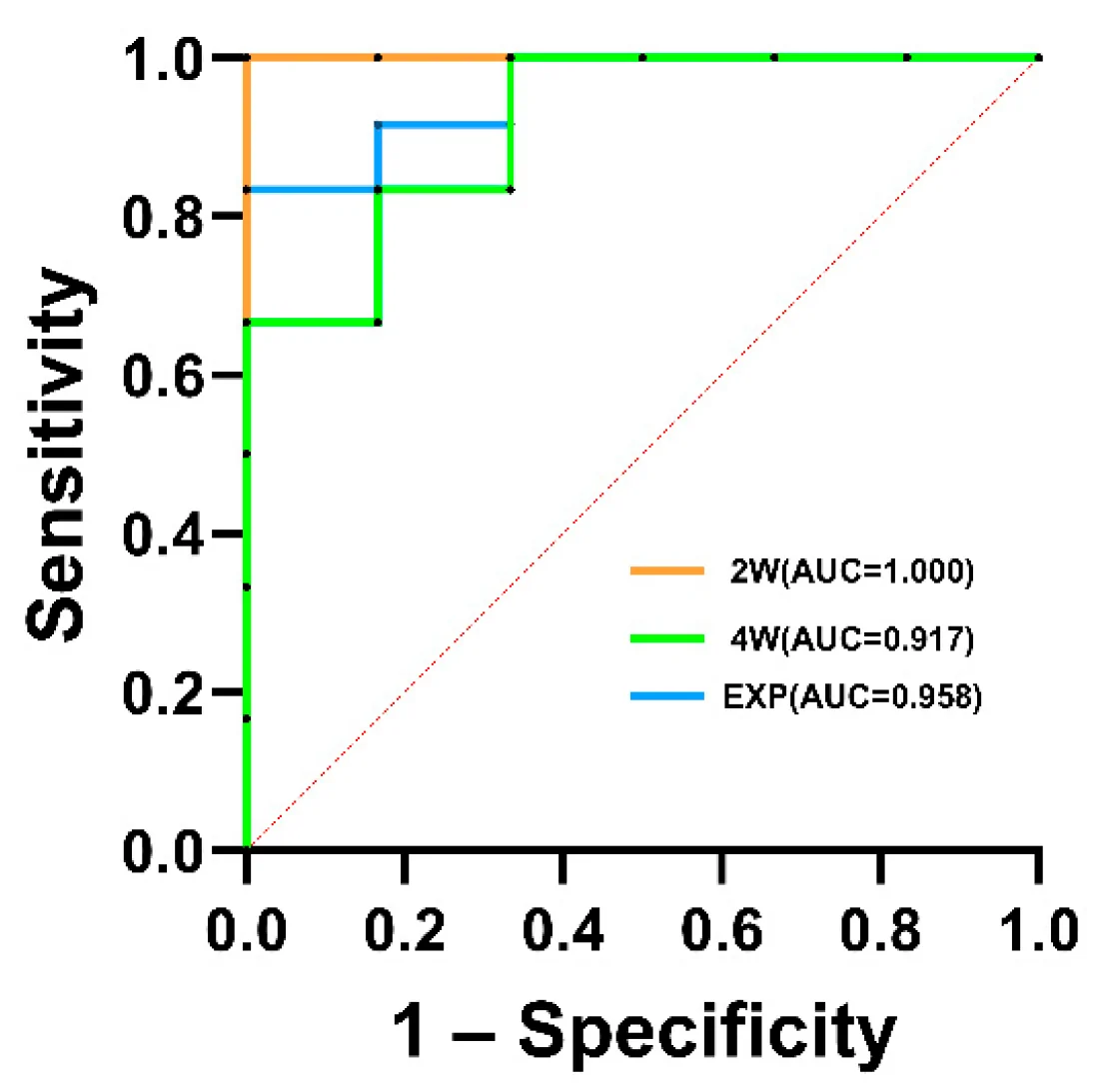
ROC curves for LTA4H. Curves for the 2-week, 4-week, and combined exposure groups against controls are shown. AUC values are displayed in the figure. The analysis should be interpreted as exploratory discrimination of exposure status. Detailed parameters are listed in Supplementary Table S4.

## Data Availability

The data presented in this study are available within the article and its Supplementary Materials. The mass spectrometry proteomics data have been deposited in the ProteomeXchange Consortium via the PRIDE partner repository with the dataset identifier PXD084316.

## Supplementary Materials

The following supporting information can be downloaded at: https://www.mdpi.com/article/10.3390/cimb48100982/s1.

## Abbreviations

The following abbreviations are used in this manuscript:

+Gz	Positive acceleration
AUC	Area under the curve
DIA	Data-independent acquisition
ELISA	Enzyme-linked immunosorbent assay
FDR	False discovery rate
IL-6	Interleukin-6
KNN	K-nearest neighbors
LTA4	Leukotriene A4
LTA4H	Leukotriene A4 hydrolase
LTB4	Leukotriene B4
ROC	Receiver operating characteristic
SD	Standard deviation
